# Decision Making in Patients With Metastatic Spine. The Role of Minimally Invasive Treatment Modalities

**DOI:** 10.3389/fonc.2019.00915

**Published:** 2019-09-19

**Authors:** Alfredo Conti, Güliz Acker, Anne Kluge, Franziska Loebel, Anita Kreimeier, Volker Budach, Peter Vajkoczy, Ilaria Ghetti, Antonino F. Germano', Carolin Senger

**Affiliations:** ^1^Department of Neurosurgery and Center for Stroke Research Berlin (CSB), Charité Universitätsmedizin Berlin, Corporate Member of Freie Universität Berlin, Humboldt-Universität zu Berlin, Berlin Institute of Health, Berlin, Germany; ^2^Berlin Institute of Health, Berlin, Germany; ^3^Charité CyberKnife Center, Charité Universitätsmedizin Berlin, Berlin, Germany; ^4^Department of Radiation Oncology, Charité Universitätsmedizin Berlin, Corporate Member of Freie Universität Berlin, Humboldt-Universität zu Berlin, Berlin Institute of Health, Berlin, Germany; ^5^Department of Neurosurgery, University of Messina, Messina, Italy

**Keywords:** spinal metastasis, stereotactic body radiotherapy, radiosurgery, minimally invasive spine surgery, separation surgery

## Abstract

Spine metastases affect more than 70% of terminal cancer patients that eventually suffer from severe pain and neurological symptoms. Nevertheless, in the overwhelming majority of the cases, a spinal metastasis represents just one location of a diffuse systemic disease. Therefore, the best practice for treatment of spinal metastases depends on many different aspects of an oncological disease, including the assessment of neurological status, pain, location, and dissemination of the disease as well as the ability to predict the risk of disease progression with neurological worsening, benefits and risks associated to treatment and, eventually, expected survival. To address this need for a framework and algorithm that takes all aspects of care into consideration, we reviewed available evidence on the multidisciplinary management of spinal metastases. According to the latest evidence, the use of stereotactic radiosurgery (SRS) or stereotactic body radiotherapy (SBRT) for spinal metastatic disease is rapidly increasing. Indeed, aggressive surgical resection may provide the best results in terms of local control, but carries a significant rate of post-surgical morbidity whose incidence and severity appears to be correlated to the extent of resection. The multidisciplinary management represents, according to current evidence, the best option for the treatment of spinal metastases. Noteworthy, according to the recent literature evidence, cases that once required radical surgical resection followed by low-dose conventional radiotherapy, can now be more effectively treated by minimally invasive spinal surgery (MISS) followed by spine SRS with decreased morbidity, improved local control, and more durable pain control. This combination allows also extending this standard of care to patients that would be too sick for an aggressive surgical treatment.

## Introduction

Metastatic involvement of the spine represents a threatening extension of neoplastic disease. Bone is the third common site of metastases in patients with systemic cancer and the spine is the most commonly involved skeletal segment (1). Postmortem examinations have shown that spine metastases affect more than 70% of terminal cancer patients. Vertebral and/or epidural(extradural) involvement is seen in 90–95% of the cases. Intradural extra-medullary and intra-medullary seeding of systemic cancer is unusual (2). Lepto-meningeal disease occurs in about 10% of patients. Symptomatic spinal metastases may be the initial manifestation of malignancy in 12–20% of cases (3). Spinal cord compression develops in 10–20% of patients with spinal disease and in 5–10% of all cancer patients (4). While pain is the most frequent symptom, 10% of cancer patients develop weakness, sensory disturbances, bowel or bladder dysfunction, and gait disturbance from instability or spinal cord compression (5, 6). In a study of over 15,000 patients with metastatic spinal cord compression, the most common histologies were lung cancer (25%), prostate cancer (16%), and multiple myeloma (11%) (7, 8). Approximately 60% of cases involve the thoracic spine, 25% the lumbosacral spine and 15% the cervical spine (9).

In the overwhelming majority of the cases, a spinal metastasis represents just one location of a diffuse systemic disease. Therefore, the best practice for treatment of spinal metastases depends on many different aspects of an oncological disease, including the assessment of neurological status, pain, location, and dissemination of the disease as well as the ability to predict the risk of disease progression with neurological worsening, benefits and risks associated to treatment and, eventually, expected survival.

Multiple prognostic scoring systems have been developed to support care providers in determining the neurological, oncological, biomechanical status of the patients as well the patient fitness, prognosis and response to therapy.

These scoring systems should be included into a framework for better decision-making in the management of spinal metastases, as well as provide a practical and reliable guidance to clinicians. To address this need for a framework and algorithm that takes all aspects of care into consideration, we reviewed available evidence on the multidisciplinary management of spinal metastases.

## Patient and Disease Assessment

The assessment of performance status, systemic burden of disease, mechanical stability, neurological risk, and eventually life expectancy is a crucial step in the selection of the best treatment for patients with spinal metastases. Based on the literature review, we analyzed how this assessment can be objectively obtained and successfully integrated into a decision making process.

### Burden of Disease and Performance Status

Assessing the prognosis of patients before treatment for metastatic spine tumor is one key point for an optimized treatment selection. A renowned method to assess the prognosis was proposed by Tokuhashi et al. (10) and Uei and Tokuhashi (11) who presented in 1989 a scoring system for the pre-operative prognostic evaluation of patients with metastatic spinal disease. A revised version has been published in 2005 (12), and the results of a prospective study which applied this revised version for treatment selection, was reported in 2009 (13). This scoring system consists of 6 items potentially influencing the outcome (general condition, number of bone metastases other than spinal metastases, number of spinal metastases, type of the primary lesion, presence or absence of metastases to major organs, and state of paralysis). According to the revised version of the scoring system, the staging of the primary lesion was changed from 3 (0–2) to 6 (0–5) levels, and the survival period was predicted to be ≤6 months when the total score was 0–8, ≥6 when the total score was 9–11, and ≥1 year when the total score was ≥12 (Table 1).

**Table 1:** Revised Tokuashi Score.

**Predictive factor**	**Score (points)**
**General conditions (KPS)**	
Poor (KPS 10–40%)	0
Moderate (KPS 50–70%)	1
Good (KPS 80–100%)	2
**Number of extraspinal bone metastatic foci**	
>3	0
1–3	1
0	2
**Number of metastasis in the vertebral bodies**	
>3	0
1–3	1
0	2
**Metastases to major internal organs**	
Resectable	0
Unresectable	1
No metastases	2
**Primary site of the cancer**	
Lung, osteosarcoma, stomach, bladder, esophagus, pancreas	0
Liver, gallbladder, unidentified	1
Others	2
Kidney, uterus	3
Rectum	4
Thyroid, prostate, breast, carcinoid tumor	4
**Spinal cord palsy**	
Complete (Frankel A, B)	0
Incomplete (Frankel CD)	1
None (Frankel E)	2
**Total points**	**Mean survival**
0–8	<6 months
9–11	≥6 months
12–15	≥12 months

Another graded prognostic assessment introduced for brain metastases to aid clinicians in selecting the best treatment option for individual patients is the recursive partitioning analysis (RPA) (14, 15) (Table 2). Few studies have tested this classification system to identify patients who are most likely to benefit from intensive treatments for spine metastases. In 2012, Chao et al. (16) applied the RPA scoring to a cohort of 176 patients. Balagamwala et al. (15) updated the analysis based on a dataset of 444 patients and reported that patients in RPA class 1 and 2 had a good and moderate overall survival of 26.7 and 13.4 months, respectively (15). Patients in RPA class 3, however, had a poor survival with a survival of only 4.5 months. This analysis suggests that patients in RPA class 1 and 2 are candidate for an intensive procedure, not only in the salvage setting but also upfront, whereas patients in RPA class 3 are best suited for conventional radiotherapy and/or palliative care.

**Table 2:** Recursive Partitioning Analysis scoring system for patients with spinal metastases.

**RPA class**	**Description**
I	KPS ≥70
	Age ≥ 65
	Controlled primary tumor
	No extracranial Metastases
II	KPS ≥70
	Age ≥ 65
	Uncontrolled primary tumor
	Presence of extracranial Metastases
III	KPS <70

Other scales have been proposed to assess outcome. Tomita et al. (17) and Kawahara et al. (18) retrospectively evaluated 67 patients including those treated conservatively and developed a new scoring system in 2001 (Table 3). The Tomita scoring system interestingly includes the evaluation of the histology of the primary tumor as prognostic factor.

**Table 3:** Tomita scoring system.

**Prognostic factors**	**Points**
**Primary tumor**	
Slow growth (breast, thyroid, etc.)	1
Moderate growth (kidney, uterus, etc.)	2
Rapid growth (lung, stomach, etc.)	4
**Visceral metastases**	
Treatable	2
Untreatable	4
**Bone metastases**	
Solitary or isolated	1
Multiple	2
**Total points**	**Predicted prognosis**
2–4	>2 years
4–6	1–2 years
6–8	6–12 months
8–10	<3 months

According to the original data, the expected survival was 2 years or longer after en bloc excision with a score of 2–4; 1–2 years including debulking with a score of 4–6; 6–12 months with palliative decompression when the score was 6–8; and 3 months or less with supportive care when it was 8–10 (17, 18).

### Histology, Sensitivity to Radiations, and Systemic Treatments

As mentioned above, histology is one major factor in decision making for SMs. Sarcoma, Melanoma and renal cell carcinoma are considered radioresistant tumors, with an indication for primary surgical treatment in most circumstances. In contrast, very radiosensitive histologies such as myeloma, germ cell tumors, hematologic tumors and small-cell carcinoma should be treated by radiotherapy techniques with a minor role for surgical resection (19, 20).

Furthermore, due to the increasing efficacy of systemic treatments, such as bisphosphonates, immunotherapies or targeted therapies with potential effect on spine metastases, ascertaining histological characteristics of primary tumor is increasingly important. Indeed, at least in selected clinical scenarios, systemic therapy might become the first line treatment for spinal cord compression due to Hodgkin's and non-Hodgkin lymphomas, germ-cell neoplasms, neuroblastoma, breast cancer, and prostate cancer (21, 22).

### Spinal Instability

Spinal instability may cause severe disability and neurological deficits that eventually impact on survival of patients. The Spine Oncology Study Group defines this spinal instability as a “loss of spinal integrity as a result of a neoplastic process that is associated with movement-related pain, symptomatic or progressive deformity, and/or neural compromise under physiologic loads” (23). Also, the mechanical status of a metastatic spine can be graded and, therefore, included into an evaluation algorithm. One of the most widely adopted systems is the “Spine Instability Neoplastic Score (SINS)” that is a spine instability scale specific to patients with cancer (Table 4). The scoring is based on six radiographic or clinical features, with total scores ranging from 0 to 18. According to this scale, spine can be classified as stable (0–6), potentially unstable (7–12), or unstable (13–18). In a study (24) examining the use of this scoring system, interobserver and intraobserver reliability was high (0.846 and 0.886). The accuracy of this scale was satisfactory, with a 80% specificity and 95% sensitivity (24).

**Table 4:** Spine Instability Neoplastic Scale (SINS).

**Location within the spine**	**Points**
Junctional (C0-C2, C7-T2, T11-L2, L5-S1)	3
Mobile spine (C3-C6, L2-L4)	2
Semi-rigid (T3-T10)	1
Rigid (S2-S5)	0
**Pain relief with recumbence and pain with movement or loading of the spine**	
Yes	3
No (occasional pain but not mechanical)	1
Pain-free lesion	0
**Bone lesion quality**	
Lytic	2
Mixed lytic or blastic	1
Blastic	0
**Radiographic spinal alignment**	
Subluxation or translation	4
De-novo deformity (kyphosis or scoliosis)	2
Normal alignment	0
**Vertebral body collapse**	
>50% collapse	3
<50% collapse	2
No collapse with 50% body involvement	1
None of the above	0
**Postero-lateral involvement of spinal elements (facet, pedicle, costo-vertebral joint fracture or replacement with tumor)**	
Unilateral	3
Bilateral	1
None of the above	0

*A score of 0–6 is classified as a stable spine, and no action is needed. A score of 7–12 receives a classification of indeterminate, and indicates potential instability, which warrants surgical consultation. A score of 13–18 indicates spinal instability that warrants surgical consultation*.

### Risk of Neurological Deficits

As a matter of fact, both current neurological function (i.e., signs or symptoms of myelopathy, radiculopathy, motor, or sensory deficits) and potential neurological compromise based on the amount of epidural disease or cord compression must be considered. A disease limited to bone, actually, poses relatively little immediate risk for the patient neurological status, and the treatment for this as compared to spinal cord compression is clearly different.

Assessment of the degree of epidural disease is crucial to determine the most suitable treatment. Bilsky et al. (25) proposed a systematic grading of the degree of epidural spinal cord compression (ESCC), which is now widely used amongst spinal oncologists.

**Figure 1:**
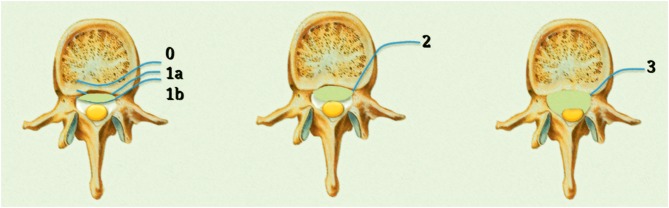
Bilski classification of epidural spinal cord compression (ESCC).

The ESCC scale consists of six grades: grade 0, bone involvement alone; grade 1, epidural impingement; grade 2, the retention of cerebrospinal fluid is visible despite spinal cord compression; and grade 3, cerebrospinal fluid is not visible due to marked spinal cord compression. Grade 1 is classified into three subgroups: grade 1a, epidural impingement without deformation of the thecal sac; grade 1b, compression of the thecal sac without spinal cord abutment; and grade 1c, deformation of the thecal sac with spinal cord abutment in the absence of spinal cord compression (25) (Figure 1).

Even though this Bilsky scoring system is increasingly recognized and adopted, Oshima et al. (26) based on a retrospective analysis of T2-weighted MR studies suggested that post-operative gait function could be predicted on the basis of the circumferential ratio of cord compression (CRCC). They emphasized that nerve function depended on the grade of CRCC suggesting that spinal cord compression is not simply due to mechanical compressive force (26). Although the authors found that a CRCC of more than 50% was correlated with poor ambulatory function, in a study by Uei et al. (27), circumferential spinal cord compression and spinal deformity were seen in some, but not all, cases of severe paralysis.

In the study by Uei et al., patients with ESCC grade 1b or worse spinal cord compression at the C1-T2 level developed ASIA grade D or worse in 50% of the cases, whereas 50% of patients with ESCC grade 1c or worse at the T3-L5 level developed ASIA grade A to D paralysis (27).

## Treatment

### External Beam Radiotherapy

Radiation therapy (RT) is an established treatment for patients with SM without vertebral collapse or significant neurological deficit (28). Three-dimensional conformal radiation therapy (3D-CRT) is the standard of practice to treat bone metastases. Three-dimensional treatment planning with multiple carefully shaped fixed fields, allows to conform dose distribution to the target volume, as well as to minimize the dose to the surrounding critical tissues. In recent years, intensity modulated radiation therapy (IMRT) and volumetric modulated arc therapy (VMAT) have increasingly gained importance for the delivery of conformal therapy. Nevertheless, conventionally fractionated or hypofractionated radiotherapy should be considered a “palliative” treatment of spinal metastases due to the dose limitation by the close proximity of the spinal cord. Radiotherapy allows, to some extent, improvement in pain, neurological deficit and functional outcome (9), especially in radiosensitive tumors (29).

In the last two decades, a series of randomized controlled trials (RCTs) showed similar results in terms of pain relief when comparing a single fraction of 8 Gy and multifraction treatments, including 30 Gy/10 fractions, 24 Gy/6 fractions and 20 Gy/5 fractions (30–35). Recently, a meta-analysis of 25 RCTs provided more robust evidence for optimal RT fractionation schedule (36). Actually, patients who received the treatment in one single fraction had higher recurrent pain rates when compared to those who received fractionated treatments (20 vs. 8%, *p* < 0.001). On the other hand, single fraction was associated to improved patient and caregiver compliance, lower acute toxicity, such as nausea/vomiting, fatigue, diarrhea, and skin complications. On the other hand, single fraction was apparently associated with a higher probability of pathological fracture and spinal cord compression, but without reaching a statistical significance [odds ratio [OR] 1.10, 95% confidence interval [CI] 0.65–1.86, *p* = 0.72 and OR 1.44, 95% CI 0.90–2.30, *p* = 0.13, respectively]. However, the value of this study has been questioned because of the primary end-points heterogeneity of the parent studies (36). Furthermore, these studies concern mostly generic bone metastases. Nonetheless, an analysis of the RTOG phase III trial comparing 30 Gy/10 fractions vs. 8 Gy/1 fraction specifically for spine metastases confirmed that single fraction treatment produced less acute toxicity and a higher rate of retreatment than multifraction radiotherapy. Single fraction and multifraction RT resulted in comparable pain relief and narcotic use at 3 months (37).

Post-operative RT has been shown to improve tumor control and reduce re-operation rates (38). More recent evidence suggested that improved outcomes for metastatic spinal cord compression are actually achieved when RT follows surgical decompression (4, 39, 40). Indeed, more patients were able to walk and retained the ability to walk, maintained continence and muscle strength, while dexamethasone and morphine doses were reduced. Radiation exposure is known however to increase the risk of subsequent surgical wound complications, even after intervals of months to years (41–45). Despite the relevance of this topic, the timing between surgery and RT has not yet been investigated thoroughly. Itshayek et al. (46) recommended a minimum interval of 1 week between pre-operative or post-operative RT and surgery based on a literature review (46). When urgent surgery is indicated after RT, Ghogawla et al. (47) have reported a wound complication in 46% of cases when the procedure is done within a week from RT. Postoperative RT also influences wound healing in a time dependent manner. Radiotherapy delivered 2–3 days post-operatively significantly affects healing more than RT delivered at 5–8 days post-operatively (48–50). Most authors recommend to delay post-operative RT about 3–4 weeks, to ensure adequate wound healing (51), as well as delay elective surgery for 6 weeks to minimize wound complications when RT has been delivered pre-operatively (51–53).

Despite all these recommendations, patients who recently received RT may develop an abrupt neurological deterioration requiring urgent surgical decompression. Postoperative wound problems secondary to recent pre-operative RT can be recorded in these patients, but modern linear accelerators (LINACs) and optimized methods using arc therapy may reduce toxicity rates typical of 3D-CRT in patients receiving surgery and radiation for spinal cord compression.

### Stereotactic Radiosurgery

The concepts of high-dose delivery and conformality that are typical of single fraction stereotactic radiosurgery (SRS) could be applied to the spine in the last two decades, after a refined image-guidance system was available. Since the initial description of image-guided spinal radiosurgery, also indicated as stereotactic body radiotherapy (SBRT) using two to five fractions, a steady increase in published reports has occurred and selection criteria for spinal radiosurgery are continuously evolving. Currently available literature strongly suggests that spinal radiosurgery of non-collapsed spinal levels is associated with higher rates of tumor control, independently from histology, and lower levels of marginal failures (54–58). This body of evidence will likely change the practice of post-surgical treatment of spinal metastases, with a shift toward radiosurgery, especially for radioresistant histologies in patients with limited systemic disease. The NRG Oncology/RTOG (Radiation Therapy Oncology Group) 0631 (“Image-Guided Radiosurgery or Stereotactic Body Radiation Therapy in Treating Patients With Localized Spine Metastasis,” clinicaltrials.gov identifier NCT00922974) is a currently ongoing RCT comparing spinal SABRT with 3DCRT aimed to provide necessary evidence (59). Another ongoing study is the Canadian Study Comparing Stereotactic Body Radiotherapy vs. Conventional Palliative Radiotherapy (CRT) for Spinal Metastases (NCT02512965). This is a Phase III trial comparing a SBRT dose of 24 Gy in 2 daily fractions. Preliminary results of this dose/fraction scheme are already available (60).

**Figure 2:**
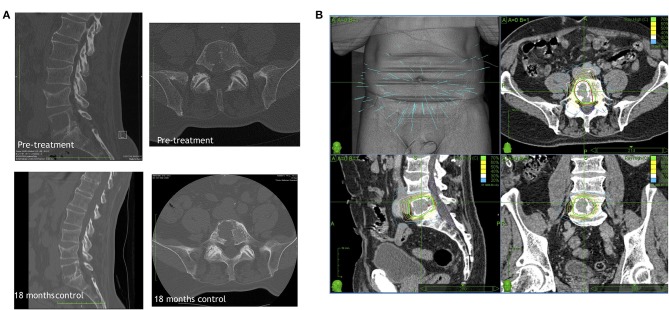
A representative primary Cyberknife treatment case showing **(A)** the CT scan of the lesion before and 18 months after the treatment visualizing the stable lesion and **(B)** the Cyberknife therapy planning.

Cyberknife image-guided robotic radiosurgery has been widely used to treat spinal metastases, also upfront (Figure 2). At the University of Pittsburgh Medical Center, in an early large clinical series, patients were treated with single-fraction radiosurgery (55). The study involved more than 500 lesions in 393 patients; lesions were secondary to different tumors, mostly moderately or highly radioresistant. The mean maximum dose to the tumor was 19 Gy delivered in single session. Sixty-seven patients had not been previously irradiated. In 48 of these cases, a significant decrease in pain was observed during the follow-up period of 6–48 months (median 16 months). Authors reported long-term local control in 88% of cases. A higher rate of local control (up to 100%) was reported for breast, lung and renal cell carcinoma when radiosurgery was the primary treatment. An overall long-term improvement in pain was obtained in 290/336 cases for whom pain was a primary indication for treatment (86%) (55).

One major problem in SRS of spinal metastases is the correct identification of the target volume when the vertebra is partially involved by tumor invasion. Patel et al. (61) evaluated the difference in clinical outcomes for patients with metastatic spine disease treated with a whole vs. partial vertebral body irradiation evaluating retrospectively 154 metastatic lesions in 117 patients. Contouring the whole vertebral body for stereotactic body radiation therapy of metastatic spinal lesions showed potential benefits by reducing the risk of recurrence, improving symptomatic relief and providing improved local tumor control. Cox et al. (62) in a consensus study proposed target volume definitions using common scenarios in intact metastatic spine radiosurgery (Table 5) (62).

**Table 5:** Summary of contouring guidelines for GTV, CTV, and PTV in spinal stereotactic radiosurgery.

**Target volume**	**Guidelines**
GTV	Contour gross tumor using all available imaging
	Include epidural and paraspinal components of the tumor
CTV	Include abnormal marrow signal suspicious for microscopic invasion
	Include bony CTV expansion to account for subclinical spread
	Should contain GTV
	Circumferential CTVs encirclincg the cord should be avoided except in rare instances were the vertebral body, bilateral pedicles/lamina, and spinous processes are all involved or when there is extensive metastatic disease along the circumference of the epidural space without spinal cord compression
PTV	Uniform expansion around the CTV
	CTV to PTV margins <3 mm
	Modified dural margin adjacent critical structures to allow spacing at discretion of the treating physician unless GTV compromised
	Never overlaps with cord
	Should contain entire GTV and CTV

*CTV, clinical target volume; GTV, gross tumor volume; PTV, planning target volume. From Cox et al. (62)*.

In a recent international consensus survey on post-operative radiosurgery practice, the gross tumor volume (GTV) was represented by the post-operative residual tumor based on MRI. The majority of participants identified the clinical treatment volume (CTV) as: the post-operative resection cavity (namely the entire extent of pre-operative tumor) plus any relevant anatomical compartment and any residual disease. The CTV margin was represented by the thecal sac rather than by the previously compressed dura. The planning treatment volume (PTV) consisted of an expansion ranging 0–2 mm. The marginal expansion in the paraspinal tissue was controversial, ranging from no additional expansion required to up to a 5-mm expansion for other clinicians. The definition of the spinal cord received a consensus considering the true cord based on either T2-weighted MRI, although the practice varies and many used a 1.5–2 mm expansion of the true cord or used the thecal sac without expansion (59).

Concerning the maximum volume suitable for spinal radiosurgery, there are reports (55) showing that spinal radiosurgery could be safely delivered to target volumes as large as 200 cm^3^. It appears that conventional volume limits of stereotactic radiosurgery could be less strict by adopting multisession treatments. However, there is a general consensus that the extent of the disease should be limited to <3 contiguous vertebral levels to be considered still suitable for radiosurgery.

Concerning the dose to be delivered, a correlation between dose and outcome has been found. Pain recurred most commonly in patients receiving <14 Gy in a single session (63). In an international survey on post-operative SRS, half of the responders used an integrated boost to areas of residual tumor. Exemplarily, for patients with radiosensitive tumors, integrated boost doses to the GTV were 16–22 Gy in a single fraction whereas for patients with radioresistant tumors, integrated boost doses to the GTV were 18–25 Gy in a single fraction or 50 Gy in 5 fractions.

Recently, Yamada et al. (64) published a very larger series of 811 vertebral metastases in 657 patients treated between 2003 and 2015 at their institution. According to results, high-dose single-session SRS provided long-term local control, regardless of tumor size, or histology. In this study, the only significant factor predictive of local control was the treatment dose. Lesions irradiated to higher doses (median GTV D_95_ of 23.6 Gy with a minimum of 18.3 Gy) had a significantly higher probability of long-lasting local control than those treated with lower doses (median PTV D_95_ of 22.3 Gy with a minimum dose of 17.4 Gy) (*p* < 0.001). The high-dose cohort had cumulative rate of local failure as low as 2% independently from histology, suggesting that this treatment modality is particularly suited for radioresistant metastases.

On the other hand, as mentioned above, the spinal cord is extremely radiosensitive. Respecting dose constraints is therefore of utmost importance. Benedict et al. (65) suggested that 10 Gy can be delivered to <10% of a spinal cord subvolume (5–6 mm above and below level treated) or to an absolute volume of <0.35 cc; a dose of 7 Gy must be limited to <1.2 cc, and a maximum dose of 14 Gy should be delivered to <0.035 cc of the spinal cord. In treatment planned in three fractions, 18 Gy is the maximal dose that can be delivered to <10% of the spinal cord subvolume or to a volume of the spinal cord <0.35 cc; a dose of 12.3 Gy is allowed to <1.2 cc of this critical organ. A maximum dose of <21.9 Gy is allowed to <0.035 cc. Sahgal et al. (66) analyzed dose-volume histogram (DVH) results for 9 cases of post- spine SBRT radiation myelopathy (RM) as compared with a cohort of 66 spine SBRT patients without RM and provided a model yielding estimates for the probability of human RM specific to SBRT (Table 6) (66).

**Table 6:** Calculated nBED (Gy_2/2_) for different probabilities of RM based on logistic regression models.

	**nBED (Gy_2/2_)**					**AUC**
	**Probability**					
	**1%**	**2%**	**3%**	**4%**	**5%**	
**Volume**						
P_max_	25.68	33.78	38.56	41.99	44.68	0.87
0.1 cc	12.88	20.79	25.46	28.81	31.44	0.83
0.2 cc	9.29	17.20	21.87	25.22	27.75	0.81
0.3 cc	6.08	14.14	18.90	22.32	25-00	0.79
0.4 cc	3.52	11.74	16.61	20.09	22.83	0.78
0.5 cc	0.76	9.26	14.28	17.89	20.71	0.77
0.6 cc	N/A	6.78	12.02	15.78	18.73	0.76
0.7 cc	N/A	4.00	9.53	13.50	16.60	0.73
0.8 cc	N/A	1.41	7.23	11.40	14.67	0.72

From Sahgal et al. (66).

*In regard to nBED 1% probability at volumes 0.6 cc or larger, algebraically they are negative doses and therefore left as not applicable (N/A). The area under the curve (AUC) for each model calculated for each volume indicates the fit of the logistic regression model*.

The steep dose fall off necessary in proximity of the spinal cord may results, however, in treatment failures. In a study from M. D. Anderson Cancer Center (54), the pattern-of-failure analysis showed two primary mechanisms of failure: (1) recurrence in the bone adjacent to the site of previous treatment, and (2) recurrence in the epidural space adjacent to the spinal cord. In-field failures occurred in 25% of recurrences and half of them in the epidural space, which was actually attributed to an underdosing of this region to maintain spinal cord constraints. This can, in specific circumstances, a major drawback of radiosurgery, which is a unique treatment modality for spine metastases and suggests the necessity of combined treatment that will be discussed later.

Another issue concerns the risk of vertebral compression fracture (VCF) after SBRT. The first major report on SBRT-induced VCF was by the Memorial Sloan-Kettering Cancer Center, who reported VCF in 27 (39%) of 71 sites treated with SBRT (67). This risk of VCF was alarming, and the median time to VCF was 25 months. Subsequently, it has been suggested in studies from the MD Anderson Cancer Center and University of Toronto that the risk may be closer to 11–20%, with a median time to VCF of 2–3 months (68, 69). The UofT, MDACC, and Cleveland Clinic pooled their clinical data specific to SBRT-induced VCF for this first multi-institutional report observed 57 fractures (57 of 410, 14%), with 47% (27 of 57) new fractures and 53% (30 of 57) fracture progression (23, 68, 69). The median time to VCF was 2.46 months (range, 0.03–43.01 months), and 65% occurred within the first 4 months. The 1- and 2-year cumulative incidences of fracture were 12.35 and 13.49%, respectively. Multivariate analysis identified dose per fraction (greatest risk for ≥ 24 Gy v 20 to 23 Gy v ≤ 19 Gy), in addition to three of the six original SINS criteria: baseline VCF, lytic tumor, and spinal deformity, as significant predictors of VCF (70).

Another point deserving attention is a potential difference of efficacy, in terms of pain control and local control, between the two regimens, single vs. hypofractionated treatments (64). As regard as EBRT, it appears that there is not a significant difference between single and multiple fraction schemes in terms of pain control. This point has been explored also for SRS: a retrospective study reported results of 348 metastatic spinal lesions in 228 consecutive patients treated at University of Pittsburg and University of Georgetown between 2000 and 2008 (71). One hundred ninety-five lesions were treated using single fraction radiosurgery (mean dose 16.3 Gy), whereas 153 lesions were treated using a hypofractionated SRT (mean doses 20.6 Gy/3 fractions, 23.8 Gy/4 fractions, and 24.5 Gy/5 fractions). The primary end point of the study was the pain control whereas subsidiary end points included: neurological function, toxicity, local control (LC), retreatment rate, and overall survival (OS).

Results turned out to be interesting and somewhat surprising. Pain control was significantly improved by a treatment delivered in one single fraction for all measured time points up to 1 year post-treatment (100 vs. 88%). Toxicity and neurological functions were not statistically different. On the other hand, the rate of LC, was significantly better in the hypofractionation group (96 vs. 70%, *p* = 0.001). Similarly, the retreatment rate was significantly lower in the hypofractionation group (1 vs. 13%, *p* < 0.001). Finally, one-year OS was significantly greater in the hypofractionation than the single fraction group (63 vs. 46%, *p* = 0.002) (71). According to the abovementioned data, whether single or multiple fraction treatments results in better tumor control remains to be further investigated. Actually, better results obtained after single fraction may simply reflect the outcomes of patients with less extensive disease (71, 72). Furthermore, most of the reported cases of radiation myelopathy reported had single fraction SBRT in the series by Sahgal et al. (66).

### Reirradiation

Salvage treatment is often necessary for patients in whom the first treatment failed. Reirradiation is a valid option, but often compromised by previous radiation to this highly radiosensitive structure. Repeated external beam radiotherapy has a risk of exceeding spinal cord constraints while reaching inferior local control compared to SRS. Maximal spinal cord doses used in clinical practice are considered to be 50 Gy for fractionated radiotherapy (TD 5/5, defined as tolerance dose with a subsequent risk of developing grad 3 myelopathy in 5% of treated patients after 5 years (73).

Clinical studies on reirradiation have usually proposed rather conservative approaches. An important RCT evaluated re-irradiation doses of 20 Gy in 5 fractions and 8 Gy in 1 fraction using EBRT techniques for painful bony metastases requiring retreatment (74). The study confirmed that the response was suboptimal with these doses, with only 30% of patients achieving an overall pain response to treatment. This clinical result highlighted the need for more effective retreatment modalities.

Modern radiotherapy techniques may have the potential to shake the universal dogma of 50 Gy maximal dose. Indeed, radiosurgery represents an appealing salvage treatment modality for patients who have already undergone EBRT yet demonstrating persistent symptoms and/or radiological progression.

The first issue of reirradiation is dose that can be used to retreat the spine. Mahadevan et al. (58) prescribed radiation doses based on the extent of spinal canal involvement; the dose was 3 × 8 Gy = 24 Gy when the tumor did not touch the spinal cord and 5 × 5 or 6 Gy = 25–30 Gy if the tumor abutted the cord. However, the choice of the dose to the spine is dependent on the dose received by the spinal cord. Thus, it is always the spinal dose constraint that should lead the selection of the dose to be prescribed to the vertebral PTV.

The largest series on the reirradiation of spinal metastases (75) reports results of 162 patients affected by 237 spinal lesions. The retreatment was performed after a median of 10.2 months from the first irradiation. The median reirradiation dose was 16 Gy in one single fraction if the first irradiation was performed using an EBRT technique and a dose of 3Gy × 10 fractions (30 Gy). Accordingly, the median tumor equivalent dose in 2 Gy fractions (the so-called EQD2) for the SRS was 34.7 Gy (considering an α/β = 10) while the median tumor EQD2 for EBRT was 32.5 Gy providing a median total tumor EQD2 of 67.2 Gy (75). Overall pain, neurological, and radiographic response rates were 81, 82, and 71%, respectively. Adverse effects occurred in 11 (6.8%) patients. Vertebral compression fractures (VCFs) were observed in 77 cases, 22 of which may be attributed to RT. Hashmi et al. reported a multicenter series of 215 patients with 247 spinal target volumes treated at 7 institutions. The median total dose/number of fractions of the initial EBRT was 30 Gy/10. The median SBRT total dose and number of fractions were 18 Gy and 1, respectively. Sixty percent of spinal target volumes were treated with single-fraction SBRT (median, 16.6 Gy and EQD2/10 = 36.8 Gy), and 40% with multiple-fraction SBRT (median 24 Gy in 3 fractions, EQD2/10 = 36 Gy). The 6- and 12-month local control rates were 93 and 83%, respectively. There were no cases of radiation myelopathy, and the vertebral compression fracture rate was 4.5% (73).

The results of these studies are in line with the dose limits proposed by Sahgal et al. (76) who suggested the use of radiosurgery for retreatment at least 5 months after conventionally fractionated RT with a reirradiation maximal dose to thecal sac corresponding to EQD2 of 20–25 Gy provided that the cumulative EQD2 does not exceed 70 Gy (considering an α/β of 2 Gy for spinal cord), and that the radiosurgery EQD2 to the thecal sac encompasses no more than 50% of the cumulative dose. Table 7 summarizes dose constraints for the spinal cord for different fractionation schemes for reirradiation according to the study by Sahgal et al. (76).

**Table 7:** Predicted maximal dose for 1–5 fractions that results in 1–5% probability of radiation myelopathy after radiosurgical reirradiation.

	**1 Fraction (Gy)**	**2 Fractions (Gy)**	**3 Fractions (Gy)**	**4 Fractions (Gy)**	**5 Fractions (Gy)**
1% Probability	9.2	12.5	14.8	16.7	18.2
2% Probability	10.7	14.6	17.4	19.6	21.5
3% Probability	11.5	15.7	18.8	21.2	23.1
4% Probability	12.0	16.4	19.6	22.2	24.4
5% Probability	12.4	17.0	20.3	23	25.3

*From Sahgal et al. (76)*.

### Surgery vs. Radiotherapy for Metastatic Spinal Cord Compression

The surgical treatment of SM has a number of potential advantages: it can actually achieve immediate decompression of the neural structures, spinal stability as well as histological diagnosis while relieving neurological symptoms (77). According to the data summarized in the previous sections of this study, indications for surgery are: evidence of neurological function deterioration or tumor progression despite radiotherapy, neurological deficit persisting after RT, radioresistant tumors, no proven cancer histology, significant metastatic spinal cord compression, spinal canal invasion, spine instability due to fracture and causing pain and neurological deficit, and a life expectancy of at least 3 months (77).

Notwithstanding these general concepts, there are some studies that help to clarify the differences of RT vs. surgery for the treatment of SM. Witham et al. (40) conducted an extensive review to compare those two treatments. Radiotherapy resulted in a mean neurological improvement of 36% while surgical management provided improvement rates of 42, 64, and 75% with laminectomy, laminectomy plus fusion, and anterior corpectomy plus fusion, respectively. On the other hand, surgical morbidity was in the range of 21–26% (78, 79), and was correlated with the extent of the surgical procedure (78) and the use of pre-operative RT (79). The mean mortality rate for posterior and anterior corpectomy were 5–6% and 10%, respectively (40).

**Figure 3:**
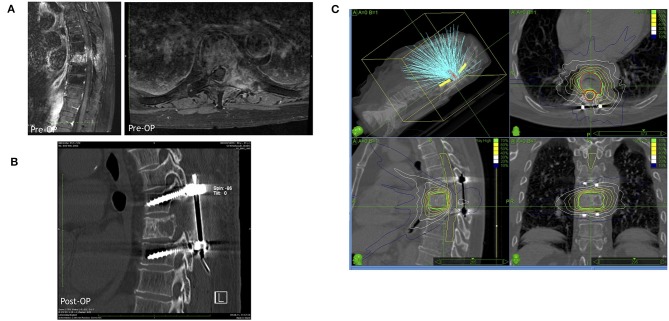
A representative case for a hybrid therapy with a prior surgery including decompression and stabilization followed by a radiosurgical treatment by Cyberknife. **(A)** Representative MRI images (left in sagittal view and right in axial view) of the metastatic lesion in thoracic vertebra 8. **(B)** Postoperative CT scan demonstrating the decompression in the lesional segment and dorsal spinal fusion of thoracic vertebra 7 and 9. **(C)** The Cyberknife treatment planning of the lesional vertebra body after surgery.

In 2005, Patchell et al. (39) published a pivotal multi-center RCT comparing surgery plus radiation with RT only (Figure 3). This study showed a clear benefit of surgical treatment for ambulation regaining or maintenance, corticosteroids intake, and analgesia. The surgical group included 50 patients who received an individualized treatment including circumferential decompression and stabilization if deemed necessary. The radiotherapy was started 2 weeks after surgery using the same treatment modality than the radiation-only group (51 patients). Ambulation was maintained by 84% of patients in the surgery groups as compared to 57% of the radiation only group. Among non-ambulating patients before the treatment, 62% in the surgical group vs. 19% in the radiation only group regained ambulation (*p* < 0.001). Patients who received also surgical decompression had an advantage in terms of neurological function, pain scores, and sphincters control maintenance. Surgical treatment warranted slightly better median survival (126 vs. 100 days). As a consequence of prolonged immobilization, morbidity was higher in the radiation therapy only group (39).

Incidentally, this study excluded highly radiosensitive tumors where the results of RT may be more comparable to surgery. Nevertheless, this is a sufficiently strong evidence to conclude that in acute paraplegia, immediate surgical debulking combined with RT is superior to RT alone (80).

A meta-analysis of non-randomized cohorts (4) similarly showed that surgery was 1.3 times more likely to maintain ambulation and twice as likely to restore ambulation. Pain improved in 90% of patients who received surgical treatment and 70% of those receiving only radiation treatment. The OS at 1 year ranged 12–62% (mean 41%) in the surgery group and 20–28% (mean 24%) in the radiation only group. Other more recent studies confirm that best results follow surgery combined with RT (4, 39, 40).

To make surgical management an effective option, surgical morbidity needs to be kept as low as possible, especially when further oncological treatment is planned. This is the rationale for the application of principles of “minimally invasive spinal surgery” (MISS) to spinal metastatic disease. Actually, MISS potentially reduces morbidity and allows earlier administration of post-operative RT and chemotherapy.

## A New Model: Minimally Invasive Surgery Plus Radiosurgery

Different approaches have been used to treat spinal metastases including: costotransversectomy, transpedicular, lateral extracavitary, transthoracic, or retroperitoneal approach based on the level of the lesions, extent of bone involvement, and surgeon preference. The aim of these approaches is to resect as much as possible of the tumor and involved vertebral levels. Nonetheless, this surgical treatment of metastatic disease has largely failed its oncological purposes. Surgery alone, indeed, cannot eradicate the disease with durable local control in most cases. In one series (81), showed that the recurrence rate at 4 years after surgery reached 96% and with no significant differences in OS between those who received complete vs. incomplete surgical resection.

On the other hand, the introduction of spine SRS into the standard treatment process allowed a paradigm shift in the treatment of metastatic disease. Actually, recent evidence demonstrates that complex surgeries provide fewer benefits than a multidisciplinary spine oncology management combined with MISS (82).

Minimally invasive procedures can be used at different stages of the patient care and include: CT-guided biopsy, local ablative techniques, cement or device vertebral augmentation, tumor embolization and, above all, microneurosurgical techniques consisting of microsurgical decompression and percutaneous pedicle-screw fixation.

Minimally invasive surgical techniques allow, indeed, the starting of adjuvant treatment as soon as 1 week after surgery, providing a significant oncological advantage compared with adjuvant radiotherapy delivered, conventionally, 1 month after open surgery to allow time for adequate wound healing.

A modern approach to metastatic spine is summarized by the concept of “separation surgery” that consists of the microsurgical restoration of the anatomical distance between the tumor and the spinal cord without attempting extensive tumor debulking and reconstruction. Separation surgery consists of a circumferential decompression of the spinal cord using microsurgical techniques. The aim of the decompression is to create a 2/3 mm corridor between the sac and the tumor to allow high-dose single-fraction irradiation of the tumor tissue minimizing the exposure of the spinal cord (83) Moreover, the spinal column is percutaneuously instrumented above and below the level of decompression to provide biomechanical stability and prevention of post-operative kyphosis (84–89). The benefits of this approach are intuitively derived from a reduced demolition of the diseased vertebral body without en bloc resection, by the limited duration of surgery and amount of surgical trauma (90).

The concept was initially introduced by a couple of studies. Moulding et al. (91) described this approach in patients who underwent decompression of the thecal sac through a posterior segmental approach followed by SRS (91). The study showed a 1-year local progression risk of 9.5%, with lower progression rates in patients undergoing high-dose single-fraction SRS. A larger study on the impact of “separation surgery” followed by adjuvant post-operative SRS was subsequently performed by Laufer et al. (83), who retrospectively analyzed 186 patients with spinal metastases and epidural spinal cord compression. The local progression rate at 1-year for single-fraction SRS was 9%, which is comparable to the results obtained by Moudling et al. (91). In an interesting study, Jakubovic et al. (92) co-registered pre-operative MRI to post-operative planning CT to delineate the pre-operative epidural GTV. The GTV was then digitally shrunk by a series of fixed amounts away from the cord (up to 6 mm) simulating incremental tumor resection and reflecting an optimal dosimetric endpoint. The dosimetric effect on simulated GTVs was analyzed using metrics such as minimum biologically effective dose (BED) to 95% of the simulated GTV (D95) and compared to the unresected epidural GTV. Epidural GTV D95 increased at an average rate of 0.88 ± 0.09 Gy_10_ per mm of resected disease up to the simulated 6 mm limit. Mean BED to D95 was 5.3 Gy_10_ (31.2%) greater than unresected cases. Accordingly, it is possible to quantify the dosimetric advantage prior to surgery (92).

One of the main surgery complication, at moderate term, is the hardware failure. It has been demonstrated that the rate of hardware failure in MISS approaches is substantially similar to that of open surgical approaches (84–89). In a retrospective study reporting data of 318 patients who received separation surgery only 9 patients (2.8%) had signs and symptoms of hardware failure requiring revision surgery (93). When looking to predictors for hardware failure, previous chest wall resection, initial construct >6 contiguous spinal levels, and female gender were associated to a significant risk (87).

Differently from open surgical treatments, MISS allows earlier post-surgical irradiation (i.e., 1 week after), with patients anecdotally treated as early as 3 days after surgery using a stereotactic technique (94). The efficacy of a combination of MISS and stereotactic body radiotherapy has been adopted also in patients with malignant primary tumors of the spine, for whom en bloc resection was not considered because of the encasement of the spinal cord or vascular structures (95).

A recent systematic review (96) analyzed results of nine studies comparing MISS and open techniques for the treatment of symptomatic vertebral metastases. In this article, the authors collected 183 patients treated by MISS that were compared with 163 patients treated by open surgical decompression and fusion. A reduced blood in MISS loss was reported by 6 studies (97–102), three described shorter operative times (97, 100, 101), four reported shorter recovery times (98, 99, 101, 102), two reported a lower complication rate (97, 101), and four reported similar or superior improvements in post-operative pain scores (97, 99, 100, 102). Furthermore, five studies showed that MISS techniques provide similar results than open surgery with regards to neurological function (97, 99–102).

Results of this review are derived from retrospective studies (9 studies provide level III evidence; 25 studies provide level IV evidence) and, therefore, their applicability remains uncertain. Nevertheless, MISS represents a promising strategy for the palliative management of spinal metastases, especially if MISS is combined with stereotactic radiosurgery. Actually, the scope of combining MISS with spinal SRS is to reduce morbidity and fast operative recovery and create a corridor between the tumor and the spinal cord for the radiation dose fall-off. As above analyzed, radiosurgical doses are more effective for local control while carry a certain risk of complications, including radioinduced myelopathy and vertebral body collapse. Separation surgery and other MISS technique clearly help preventing these risks (83, 91, 103, 104).

## Conclusions

**Figure 4:**
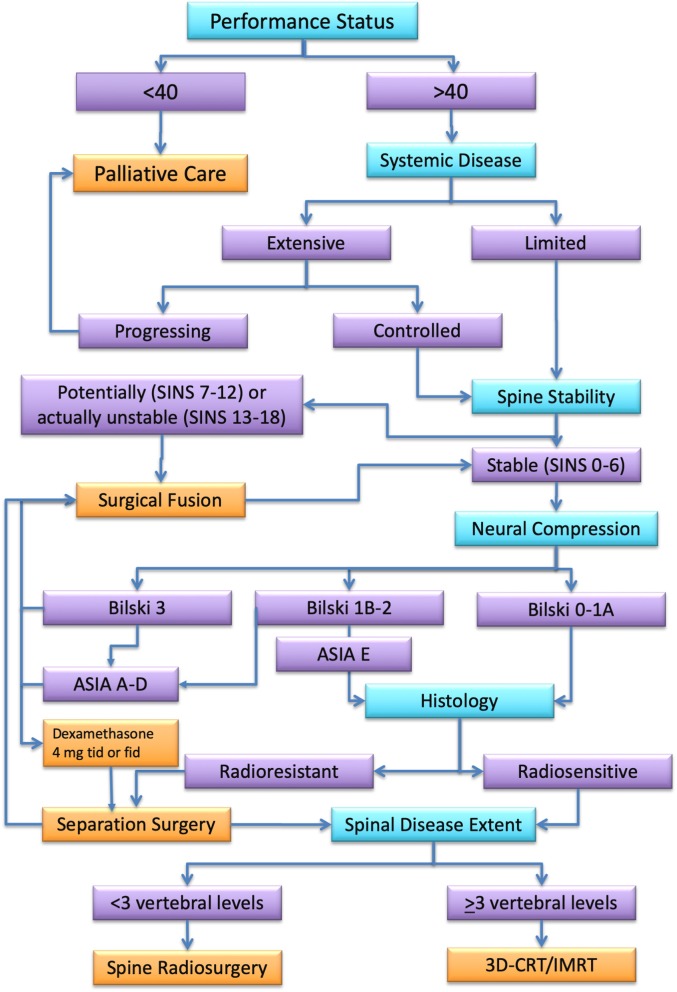
Algorithm for spinal metastasis treatment. In turquoise, the assessment steps. In orange, the best treatment option for each assessment category (in purple) according to the literature review.

Spinal metastatic disease remains a complicated multidisciplinary challenge. Early diagnosis is essential, because treatment outcome depends on pretreatment neurologic function. The use of SRS/SBRT for spinal metastatic disease is rapidly increasing. Aggressive surgical resection may provide the best results in terms of local control. Nonetheless, it carries a significant rate of post-surgical morbidity whose incidence and severity are correlated with the extent of resection. Complications and amount of surgical impact on oncological patients often cause a delay of adjuvant treatment that, indeed, abolishes any advantage of an aggressive surgical management. A multidisciplinary management represents, according to current evidence, the best option for the treatment of spinal metastases. Figure 4 summarizes an algorithm for multidisciplinary management we have drawn on the base of available clinical data. Noteworthy, according to evidence, cases that once required radical surgical resection followed by low-dose conventional radiotherapy, can now be more effectively treated by separation surgery and spine SRS with decreased morbidity, improved local control, and more durable pain control. This combination allows also to extend this standard of care to patients that would be too sick for an aggressive surgical treatment. Future efforts are needed to prospectively compare the effectiveness of these available treatment approaches, focusing primarily on outcomes of tumor control, treatment-related morbidity, and quality of life.

## Author Contributions

AC analyzed data, wrote the manuscript, and approved the final version.GA, AKl, FL, AKr, and CS contributed to Pubmed search, manuscript writing, and figures. VB, PV, IG, and AG revised the manuscript.

### Conflict of Interest Statement

The authors declare that the research was conducted in the absence of any commercial or financial relationships that could be construed as a potential conflict of interest.
